# Experiences of Norwegian co-parents during COVID-19 pandemic restrictions: A qualitative study

**DOI:** 10.1080/17482631.2023.2215578

**Published:** 2023-05-23

**Authors:** Heidi Jerpseth, Anne Kaasen, Sara Rivenes Lafontan, Tine Schauer Eri

**Affiliations:** Faculty of Health Sciences, Department of Nursing and Health Promotion, Oslo Metropolitan University, Oslo, Norway

**Keywords:** Co-parents, experiences, maternity care, COVID-19 pandemic, thematic analysis

## Abstract

**Purpose:**

The COVID-19 pandemic restrictions have had a major impact on the organization of health services in Europe. Co-parents’ experiences of not being allowed to fully participate during pregnancy, childbirth, and the postpartum period is poorly understood. We investigated how the non-birthing partner experienced becoming a parent during the pandemic.

**Methods:**

We applied a qualitative design. We recruited the participants from all part of the country by using snowball sampling. 18 individual interviews were conducted by using videotelephony software program/telephone. The transcripts were analysed using a six-step model for thematic analysis.

**Results:**

The non-birthing participants were not considered by the healthcare system to be equal partners in terms of their involvement in the process of becoming parents. Three themes were constructed from the interview analysis- deprivation of the opportunity of “doing their part” of the job; participation by proxy to enhance togetherness; and choosing between obedience or opposition to the restrictions.

**Conclusion:**

The non-birthing co-parents felt deprived of doing what they considered to be their most important job—namely, to support and comfort their partners during pregnancy and childbirth. The healthcare system’s decision to exclude co-parents from being physically present thus requires further reflection and discussion.

## Introduction

Over the past two years, the COVID-19 pandemic has had a major impact on how health services during pregnancy, childbirth, and the postpartum period in Europe have been organized to reduce infection (Coxon et al., 2020). During the COVID-19 pandemic in Norway, as in many other countries, restrictions were imposed to prevent the spread of infection. Several of these restrictions greatly reduced the access of non-birthing partners to health facilities during pregnancy, childbirth, and the postpartum period (Coxon et al., 2020; Renfrew et al., 2020).

Partners were not allowed to attend pregnancy consultations or be present at the birth until active labour^1^ was well established. Additionally, they were often not permitted to be present during the post-natal stay in hospital. Restrictions led to limitations on visits, including visits by the parents’ own children, during hospital stays (Eri et al., 2022; Naurin et al., 2021). However, restrictions differed across the country, with health facilities in urban areas implementing the most severe restrictions.

There has been momentous change in non-birthing partners’ participation in all events involved in having a child. Some decades ago, they were not allowed in the delivery rooms (Condon et al., 2004; Opondo et al., 2016). Given major changes in society, the organization of health facilities, and the disunity of labour within families, the partner’s role has changed from being that of a slightly distant participant in terms of pregnancy, birth, and infant care to be a more active participant (Brunstad et al., 2020; Ellberg et al., 2010; Redshaw & Henderson, 2013). In Norway, the non-birthing parent is considered to be an integral and equal part during the birth of the baby and the stay in hospital after birth. The partner is also recognized as an important caregiver to the new-born, and 15 weeks of the 45 weeks of paid parental leave is exclusively for the non-birthing partner. This part of the leave cannot be transferred to the mother unless she is a single mother caring for the child alone which is stated in the White paper (Meld. St., 2008–2009). Childbirth has been described as a mutually shared process for the couple, and the non- birthing partner’s recognition as a parent should also be supported (Premberg et al., 2011).

Family formation in the sense of having a child has been recognized as a significant transitional phase in life (Condon et al., 2004). The importance of both birthing partner and the non-birthing partners’ involvement in the process has also been shown to be essential for how the couple lives through this transitional phase and are strengthened by the process. It has been argued that how the family experiences having a child and how the mother and partner cope with this transition is vital for the family’s health in the long term (Opondo et al., 2016). Studies have shown that the co-parents’ involvement and participation in the whole process of pregnancy, labour, and the postpartum period are important for the future health of the mothers, co-parents, and children (Erlandsson et al., 2007; Latifses et al., 2005; Opondo et al., 2016; Raby et al., 2015; Wolfberg et al., 2004). The engagement of the partner may influence a pregnant birthing partners’ perception of care, uptake of services, and maternal outcomes (Redshaw & Henderson, 2013). However, non-birthing parents sometimes feel like they are present but invisible during this critical time in the lives of both parents and newborn children. This is concerning, as research has shown that partners’ expectations, and the violations of these expectations throughout the transition to parenthood, greatly impacts the relationships within couples (Hodgson et al., 2021; Mitnick et al., 2022). Additionally, standardized guidelines for involving co-parents in perinatal care are lacking (van Vulpen et al., 2021). As shown above we have significant knowledge about the importance of the co-parents’ presence in the whole process of pregnancy, labour, and postpartum period. In this perspective we will expect that the pandemic restrictions have had a detrimental impact on families. In this perspective we want to gain more knowledge about co-parents’ experiences of becoming parents during the COVID-19 pandemic.

The aim of this study is to describe how the non-birthing partners experienced becoming parents during the pandemic, with a focus on pregnancy, birth, and the immediate postpartum period. The following research question guided the study: How do partners of women who have given birth during the COVID-19 pandemic experience their role, their participation, and the organization of the healthcare system?

## Methods

### Reflexive note on the authors

All authors have a background from clinical and academic midwifery or nursing. We are all concerned about families having the best possible start and believe that this can impact the family formation in the short and long run. We are concerned about how the time of the COVID-19 pandemic and the restrictions have affected parents and families; however, we have no direct experiences of this ourselves.

### Design

We applied a qualitative research design comprising individual interviews. In this study, the experiences of partners of pregnant women were of particular interest due to the extraordinary situation caused by the COVID-19 pandemic restrictions. The restrictions reduced the possibilities for the partners to take part in antenatal care, during labour and birth, and postpartum care. We used a thematic analysis with a six-step model inspired by Braun and Clarke (Braun et al., 2022) to analyse the data.

### Setting

Norway’s maternity care system, part of the tax-funded public healthcare system, serves virtually all women in Norway and is provided free of charge at delivery. Private care is rare, but there are private clinics offering ultrasound scans, as well as a few independent midwives offering pregnancy care and homebirths. Most women receive antenatal care from a combination of midwives and general practitioners in the municipalities. Intrapartum care is organized according to the following three levels: specialized obstetric units, obstetric units, and birth centres (alongside obstetric units or freestanding). Due to geography and demography, the population is widely scattered, and maternity services are characterized by both centralization and decentralization. In this paper, the father or co-mother is referred to as either “the partner,” “the co-parent,” or the “non-birthing partner.”

### Recruitment and participants

We wanted to gather a wide variety of voices from the target population. We included participants from the four health regions in Norway (South-Eastern Norway Regional Health Authority, Western Norway Regional Health Authority, Northern Norway Regional Health Authority, and Central Norway Regional Health Authority), to capture experiences with various degrees of COVID-19 restrictions. Snowball sampling was considered suitable. In this recruitment strategy, researchers recruit people they know to participate in the study; those new participants then suggest people they know for inclusion in the study, and so on (Tenzek, 2017). In the present work, the researchers used their own networks and contacted potential participants in the target population, asking them to participate in the study. Personal contacts provided the initial introduction to the study for one co-parent and snowball sampling proceeded from them. After the interviews, we asked the participants if they knew someone that could be interested in participating in this study and asked them to forward our contact details. This process continued (i.e., like a snowball), and we chose to set a limit with 18 participants, which was considered beneficial for covering a nuanced and compound picture of experiences in addition to ensuring geographical variation among participants (Malterud et al., 2016).

The inclusion criteria included partners to women who had given birth during the period since the outbreak of the COVID-19 pandemic from March 2020, with a positive foetal outcome. The inclusion criteria did not specify whether it was a first-time parent or not. Eighteen co-parents were included in the study, seventeen men and one woman. The age of the participants ranged from 27 to 46 years. Ten of the participants were first-time parents, seven were parents for the second time, and one for the third time. The ages of the children born during the pandemic varied from one month to one and a half years when the interviews were conducted.

### Data collection

The data collection consisted of individual interviews based on an interview guide. We used a videotelephony software program, Zoom to implement the interviews, except one which was conducted by telephone. We asked the participants to narrate their experiences from the beginning of pregnancy to maternity leave. Their experiences regarding being a partner and expecting a child during the pandemic were explored via various questions, including the following: Can you tell me about how you were present during the antenatal visits? How did you feel about being present/not being present? When looking back, in what way do you think that the pandemic-related restrictions have influenced your experiences of becoming a parent?

The interviews lasted between 30 and 60 minutes and were transcribed verbatim but de-identified by a professional language editor. The data collection took place between June and August 2021.

### Data analysis

We used a six-phase analytic process inspired by Braun and Clarke (Braun et al., 2022) to identify patterns and themes. The phases are referred to; (1) Familiarisation with the data. (2) Generating initial codes. (3) Generating themes. (4) Reviewing themes. (5) Defining and naming themes. (6) producing the report (Braun et al., 2022). We carried out the process of coding the material in a flexible way and moved back and forth through the phases as necessary (Braun & Clarke, 2019, 2021b). In the first phase, the first author listened to the interviews and wrote down comments in the transcribed texts. All the authors read and reread the transcripts to familiarize with the data. We organized meetings wherein we wrote down our first impressions of the dataset and discussed the notes and why we had these first impressions regarding the meaning of the dataset. Through this reflexive process, we tried to avoid predefining themes before coding, but we suggested possible interpretations of the material. Then, in the next phase, the first and the last author carried out more detailed and systematic work and extracted the meaning content from the data and generated codes. We used open thematic coding for each transcript and searched for patterns or themes within the data. In the third phase we decided which codes fit together and manually constructed initial themes. Themes were identified and discussed across the data and in line with the aim of the study and our own interpretations (Table I). In the fourth phase, all authors discussed the initial themes, and modified the themes to avoid overlaps. In the fifth phase, all authors reread the original data to check the themes and quotations, ensuring that the themes authentically embraced the content of the interviews. In the final phase, we checked how well the themes worked, together and individually, and ensured that the themes provided a coherent description corresponding to the aim of the study. All the researchers were involved in and agreed on the summarized themes shown in the findings.

**Table I.** Illustrations of how data were structured into themes.

Data extract	Codes	Themes
*There were not that many ways I could contribute. By being there and providing practical support and stuff like that. But that was in a way taken from me. I wanted to be the best possible father, which means being there during the entire process*	Wanting to help Not being able to be the supporting partner Stressful and difficult to handle	Deprivation of the opportunity to “do my part of the job”
*We solved it in different ways. For example, when we were to learn the gender of the baby, we asked the midwife to write it on a note that we could open and read together. I was outside and waited and she came out and then we opened the note*	To achieve altered togetherness To experience as much as possible of the process together as a couple	Participation by proxy to enhance togetherness
*I went home after the delivery because I wasn’t allowed into the postnatal ward. It was painful to leave her because she was at her most vulnerable and the ward was very busy. You’re punished for being obedient since you don’t want to cause any trouble. Should I have had a different role? Should I have raised it with staff?*	Experienced feelings of guilt and shame for being too obedient and cooperative Not being more loyal to their own wishes and meeting their partners` needs to have them nearby	Choosing between obeying or opposing restrictions

### Ethical considerations

The study was approved by the Norwegian Centre for Research Data in June 2021 (nr.881542). All the participants gave their written, voluntary consent to participate in the study. The participants were informed about their right to withdraw from the study at any time during the research process before the analyses. All participants received an email providing information about the study. We ensured that the researchers did not know the participants being interviewed. The participants agreed to our use of a videotelephony software program, Zoom (Santa Clara, Ca., USA) (with exception of one participant who did not have Zoom installed, and the interview was instead conducted by telephone). We used the app connected to Services for Sensitive Research data to record the interviews (Nettskjema—University of Oslo [uio.no]). The sound files were password protected, and only researchers were allowed to access the files. The data was anonymized and stored in accordance with the current guidelines at the Oslo Metropolitan University and in accordance with the General Data Protection Regulation (GDPR).

## Findings

The participants experienced worry and uncertainty during the pregnancies due to the restrictions, which differed both geographically and over time during the pandemic. They were determined and wanted to share the responsibility with their partner, being the person on whom their partner could depend on throughout the pregnancy, labour, birth, and postpartum period. It was important for the participants to partake in the whole process in their own transition to parenthood, to create their own identity as co-parent. However, they experienced being partly deprived of this opportunity during the pandemic. To cope with the restrictions, they found other ways to equally contribute to the experience both in terms of *time* and *distance*. The following three themes were identified: deprivation of the opportunity to “do my part of the job”, participation by proxy to enhance togetherness, and *choosing between obeying or opposing restrictions*

### Deprivation of the opportunity to “do my part of the job”

The partners characterized their own role partly as the one that should support, facilitate, and contribute practical things. However, they also characterized their own role as autonomous, by highlighting their own significance in being a co-parent. As the pregnancy progressed, the participants became increasingly worried about not being allowed to be present during labour and birth, which they considered their most important job. This concern was both related to the ever-changing COVID-19 restrictions, and their own fear of becoming infected just before the due date. The period leading up to the due date was described as very tense:

We did all that we could to avoid being around other people. There were only the two of us during the last three weeks before the due date (13).

The participants characterized the ever-changing rules to limit the spread of COVID-19 as frustrating. They used different strategies to try to make the unpredictable more predictable. Some read the hospital’s information on the website several times a day to update themselves regarding the changing restrictions. Others focused on reducing the number of people whom they came in contact as much as possible in fear of being infected, even if it meant changing their normal way of working:

It was difficult to feel in control of the situation. We had no idea which restrictions would be in place at the hospital. We heard of couples who were not allowed to be together during labour and birth. It worried me a lot. I planned at work to avoid meeting clients and we did not meet with friends and family. (4)

All the co-parents praised their own partners and considered them to be the ones who did the most important work. Not being able to be the supporting partner as much as they wanted to, was characterized as stressful and difficult to handle:

There were not that many ways I could contribute. I cannot just get pregnant myself. But I can support her and make sure the pregnancy and delivery are as smooth as possible for my wife. By being there and providing practical support and stuff like that. But that was in a way taken from me. I felt deprived of how little I could contribute with. I wanted to be the best possible father, which means being there during the entire process (18).

Due to the restrictions limiting the presence of the non-birthing partner during antenatal visits, most of the participants had scheduled one ultrasound examination at a private clinic. In the private healthcare sector, there were no restrictions for the co-parents’ presence. To be present during one ultrasound examination was especially important for the participants who were expecting their first child. They experienced this examination as a turning point in their manifestation of becoming a parent:

We went to a couple of ultrasound scans at a private clinic so that I could be there and watch it “live.” We saw the foetal heartbeat; it was incredible, and we got to experience it together. It made the situation easier. (8)

At the maternity units in the hospitals, the restrictions dictated that the co-parent was not allowed to enter the hospital before the midwife had assessed the birthing partner to be in the active phase of the first stage of labour. Throughout, the findings of our study shown that the co-parents’ concerns and preoccupations were linked to their perceived role as being an invaluable and important support for their birthing partner. Not being able to be the supportive partner they wanted to be was characterized as stressful and difficult to cope with:

It was difficult to hear on the phone how much pain she was in and how distressed and scared she was without [me] being allowed into the hospital. It was very painful not being allowed to comfort her. (11)

Some of the participants felt deprived of an important milestone in their lives because they missed their chance to participate alongside and support their partner during the whole maternity episode. The non-birthing parents perceived themselves and their partners as a team. During pregnancy, most of the couples had planned how they wanted to interact in the process of becoming parents. This interaction meant, among other things, that in addition to being a support for their pregnant partners, the co-parents also wanted to be something beyond simply being a “support person.” They aimed to have an autonomous role in parenthood as well. However, some of the co-parents talked about the attitudes both of their own parents and healthcare personnel questioning their importance in pregnancy, labour, and postpartum, which has traditionally been considered a women’s domain:

The role of the co-parent has traditionally been considered as less significant. This attitude is not good for becoming a family (16).

### Participation by proxy to enhance togetherness

This theme involves the diverse ways the participants both directly and indirectly participated in the processes of pregnancy and the birth of their child, as well as the various strategies they developed to try to make such participation possible. The partners experienced the COVID-19 restrictions as affecting their participation and involvement in pregnancy, labour, birth, and the postpartum period in various ways depending on time during the pandemic at which the birth occurred and the local restrictions. If not able to be present, the main strategy was to stay as close as possible—both in relation to *time* and to *distance*. This could mean following the pregnant birthing partner to the health clinic for pregnancy consultation, waiting outside the clinic during the consultation, and being immediately present when she came out. This way, the non-birthing parent felt a kind of physical presence, and it contributed to reducing the feeling of distance. Additionally, the couples discussed what they would like to gain information about shortly before they had to separate for a given appointment; then, the pregnant birthing partner would share the information obtained and the significant impressions from the consultation with the co-parent as soon as possible afterwards. This strategy was reported to enhance the *proximity* when the non-birthing parent was not able to participate directly. The feeling of proximity was important in relation to the participants’ own feelings of being involved in the process, but also because they wanted to be present to support the birthing partner, if they were in need for further consultation. One participant who had waited outside the health facility during antenatal care visits explained the following:

Well, it was about being there in case there were any bad news and could … for us to be able to meet as quickly as possible, which was important for me (1).

However, to be waiting outside the healthcare clinic or the hospital could be unpredictable in terms of duration and could end up taking hours. Partners reported waiting outside in a car or on a bench for several hours without information about progress. On these occasions, the strategy of enhancing proximity made the partners feel unease. The lack of information and the long waiting times instead contributed to feelings of uncertainty and loneliness:

It was quite nerve-wracking to wait outside in the car. Okay, what does it mean that the amniotic fluid is coloured? We didn’t really know a lot if anything had gone wrong by then. So, it was quite scary to be outside and not know knowing like … it was actually a pretty scary experience. I tried to pass the time there in the car, but it wasn’t easy (8).

The various strategies were also a means to achieve *altered togetherness*—to experience as much as possible of the process together as a couple and as parents-to-be, but in a different way because of the pandemic-related restrictions. For many participants, the moment they heard the heartbeat of the foetus for the first time or saw the ultrasound scan when the foetus’s gender was revealed, were pivotal moments they really wanted to share with their co-parent. One partner described how he waited outside the hospital in the parking lot during the ultrasound scan and how the gender of the foetus was not revealed until they were both together in the car:

We solved it in different ways. For example, when we were to learn the gender of the baby, we asked the midwife to write it on a note that we could open and read together. I was outside and waited and she came out and then we opened the note. In a way, we got to experience some of those moments together but in a completely different way (1).

Yet another strategy for participation and involvement during the process was to take advantage of the opportunities offered by *technology*. This could involve recording the consultations with the midwife throughout pregnancy and then listening together and discussing afterwards. Furthermore, some of the participants took a video of the ultrasound scan performed by the healthcare professionals while they explained everything for the camera. These actions could lead to the partners feeling more involved and active as a parent and enhance the sense of proximity to the actual situation. However, if this happened at pivotal moments such as when the gender of the foetus was revealed, it could also lead to more negative feelings, thereby reducing proximity and togetherness:

When we learned the gender, I was in the car outside and on FaceTime, which was pretty strange (2).

Furthermore, the theme of participation by proxy points to the limited time the partners were allowed to be present when the birthing partner was in the hospital; the amount of time the partner spent alone during this period far exceeded the amount of time they spent together. This could be before labour, for example, if the birthing partner was induced, during labour, when the partner’s presence was restricted, or after labour, when the mother and child were in the postnatal ward. The partner experienced being reduced to a visitor with no rights of their own and not the co-parent they wanted to be:

I received a phone call saying that visitors were allowed, that I was allowed to visit during visiting hours. I was to leave afterwards but I could stay for about one hour. It was the usual visiting hours between 5 pm and 7 pm or something like that. Afterwards, I would go home and return when the labour started (7).

### Choosing between obeying or opposing restrictions

Some participants were unsure whether they used the right strategy by following the restrictions to control the infection, or if they should have opposed the rules in some situations. They blamed themselves for not being more loyal to their own wishes and meeting their partners’ needs to have them nearby. Some participants experienced feelings of guilt and shame for being too obedient and cooperative. In hindsight, they reflected on whether they should have taken the risk and stayed with their partner without asking anyone or even claiming their right to be present. However, the participants’ main concern was that they missed being supportive to their birthing partner and advocating for their partners to ensure their wellbeing:

So, I went home after the delivery because I wasn’t allowed into the postnatal ward. It was painful to leave her because she was at her most vulnerable and the ward was very busy. You’re punished for being obedient since you don’t want to cause any trouble. Should I have had a different role? Should I have raised it with staff? I’ve heard of fathers who have made a scene and got it the way they wanted it, which is difficult for me. But making a scene and causing trouble is not who I am as a person. Should one be ashamed of not being able to take care of one’s partner because you are not one of those people who shouts the loudest to get what you want? (16)

Other participants used the opposite strategy and simply walked into the hospital without saying anything or stated that it was not an option that they should wait in the car or not be present at the hospital.

The midwives were hesitant, but I called and said that I was coming immediately. They probably understood that I was not going to give in. So, they bent the rules (12).

The participants struggled to understand the logic of some of the restrictions and believed that some did not make sense. They pointed out that living with the pregnant partner meant that they would both be infected with COVID-19 if one of them became infected. In this case, their presence during an examination or hospitalization did not pose an increased risk. Some of the partners also found it difficult to understand the rationale behind being treated as a visitor. As a visitor, they were allowed two hours a day to visit their partners and newborn child, which meant that they were living their normal lives when they were not visitors—taking the bus, shopping, working, and exposing themselves to possible infection. All they wanted was to stay in the hospital together with the birthing partner and their newborn child:

If the mother had been infected, it would have been because of the father, and if the father was infected, the mother would have been, too. It doesn’t make sense. If they believe that the mother, father, and baby is a threesome, it is going to get very lonely to be a father who is not allowed to be present. That feeling of powerlessness by not being able to contribute. I was not allowed to be there and provide support. (18)

Other participants found the restrictions sensible and understandable. They accepted the premise and did not question the decisions, even though the restrictions limited their own presence. By accepting the rules, they believed that they were a part of something more significant than their own participation. They wanted to contribute and to take responsibility for the situation that the whole society was in due to the pandemic:

I accepted the rules and understood the rationale behind the restriction. Nothing seemed unreasonable. It was a loss, but it was my duty to abide by the rules (3).

The participants expressed mixed feelings about the experience of becoming a parent during a pandemic. Almost all the participants saw their own situation as a dichotomy; on one side, they were grateful and happy because of their child, on the other side, they felt sidelined in a situation described as the most important event in their lives. These feelings of gratefulness extinguished feelings of disappointment and bitterness. Some of the participants also felt sad and angry, which in turn gave them feelings of guilt, as they had just experienced something as important as having a child.

I had to leave after the delivery. It sucked but was also nice because it created calm for my wife and child. If you look on the bright side, it was good for the two of the them where she could experience becoming a mother. By framing it like this it is easier for me not to be bitter because (the restrictions) were important for our society. I was above all very grateful. (13)

Even though the participants felt grateful more than anything else, some of them reported the experience as something that they had looked forward to but did not happen in the way they had expected. They tried to cope with feelings of disappointment and did not know towards whom this feeling should be directed:

I did not know where to direct this bitterness. At the same time, I noticed that I was getting very annoyed with the guards responsible for letting us in during visiting hours. They became the manifestation of who was in charge. They scolded me because I had misunderstood the visiting hours and had to wait one hour outside the hospital before being allowed inside to see my own wife and child. It was in a way humiliating. (16)

## Discussion

The main findings highlight the participants’ experiences of not being considered an equal partner in terms of their participation in the process of becoming a parent. They were not perceived as an integral part in perinatal care, and instead were reduced to being a visitor. This exclusion led to feelings of insecurity, stress, worry, and even shame. The participants’ main fears were linked to their concerns regarding the welfare of the mother and child, as well as to their own needs to be a part of or share in the experiences of becoming a parent. The participants strove to find predictability in a time characterized by unpredictability both related to the restrictions and the progression of the pandemic. They enhanced togetherness in ways other than those they had planned. However, some participants struggled with thoughts of whether they were too obedient following in the rules that kept them away

The participants experienced that their expectations of being physically present had a double meaning in the process of becoming a parent. They evaluated their main job as being a support to the mother; however, they also highlighted the importance of being present for their own sake. They were disappointed with and in despair at finding themselves in a situation that was unpredictable and different than what they had expected and hoped for. Although they developed various strategies to be present and stay as close as they could, they experienced that their opportunity to be a team was disrupted. One common strategy for the participants in this study was to visit a private clinic so that both partners could be present at the ultrasound screening. The co-parents evaluated this opportunity as important to their experience of being a part of the process of becoming parents together with the birthing partner. Listening to the heartbeats and watching the foetus on ultrasound screen is important for prenatal bonding (Walsh et al., 2014; Wells, 2016). However, ultrasound examination at a private clinic is quite expensive, and thus this opportunity was not a possibility in the population at large.

The private ultrasound examination was a onetime occurrence for most of the participants; during the rest of the process in perinatal care, the non-birthing parent was partly excluded from physical presence. To be physically absent from birth and postnatal care can entail a sense of isolation, which can lead to reduced bonding both with their partner and child (Nespoli et al., 2022; Sweet et al., 2022). One literature review showed that the non-birthing parent tends to bond best through participation in birth and postnatal care (Scism & Cobb, 2017).

The World Health Organization’s (WHO) recommendations highlight the importance of the co-parents’ active participation during pregnancy, birth, and early parenthood as a key component of providing respectful care. The WHO stated that during the pandemic, all childbearing women must have the opportunity to have companionship during the pregnancy, labour, and birth (World Health Organization, 2020).

Additionally, studies have shown that labouring women with continuous support of a co-parent are more likely to give birth spontaneously with less use of pain medication, have slightly shorter labours, and give birth to newborns who are less likely to have low five-minute Apgar scores (Bohren et al., 2019; Erlandsson et al., 2007; Latifses et al., 2005; Opondo et al., 2016; Raby et al., 2015). A family-centred approach is not new; however, healthcare providers seem to be continually unsuccessful in involving co-parents in pregnancy and postnatal care (Ellberg et al., 2010; Feenstra et al., 2018; Johansson et al., 2015; Steen et al., 2012). This might indicate that the practice of not including the co-parent is not specifically related to the restrictions during the pandemic and rather that healthcare professionals consider the non-birthing partner to be outside of the process of the immediate becoming a family. There seems to be a need to strengthen the inclusion of the non-birthing parent in national guidelines to bridge the gap between policies and practice in this area.

Furthermore, women want a companion during labour and childbirth. They want to be with somebody they trust and someone who is compassionate (Vedeler et al., 2022). In short, companionship might produce and reinforce a positive birth experience, and excluding co-parents from participating in perinatal care might have consequences beyond the co-parents themselves (Bohren et al., 2019; Opondo et al., 2016; Xue et al., 2018). This need for support and compassion by the birthing parents was even more present throughout the pandemic, and studies have underlined that these mothers felt insecure and lonely, as well as missed their partners in a situation where they would typically be together (Ceulemans et al., 2021; Eri et al., 2022; Naurin et al., 2021). This coincides with the co-parents’ view on their own role and assessment of their importance in this present study. The co-parents wanted to be present during the entire process, and they regarded their role as being the one who should be compassionate and trustful. The participants suffered when they experienced that the birthing partner needed them to stay close, but they were forced to stay apart. This is in line with one study that showed that co-parents’ main worries were related to the safety of the pregnant birthing partner and their unborn child (Naurin et al., 2021). The co-parents’ involvement during the perinatal period is therefore necessary for both birthing and non-birthing parents to reduce anxiety, and promote foetal-maternal attachment (Wells, 2016).

The participants’ experience of shame was caused not only by failing to stand up for the birthing partner who needed them to be present but also betraying themselves regarding their own rights and wishes to be involved in the process of becoming parents. Shame is a complex emotion arising from the sense that something is fundamentally wrong about oneself. This means that shame is linked to a person’s being or identity (Miceli & Castelfranchi, 2018). The co-partners’ experiences of shame might be reinforced by their experiences of not being acknowledged by health care professionals. They experienced that some healthcare professionals did not seem to understand what was at stake for the co-parents and underestimated the couples’ need to be together in a vulnerable situation. Subsequently, the participants learning about other partners who had claimed their right to be present might have reinforced their own feelings of shame for not being more assertive on behalf of themselves and their partner. Although many of the participants had trust in public health authorities and agreed with the restrictions to control the pandemic, some of the participants found it difficult to understand the logical reasons for being left outside with limited understanding and information regarding the reasons for the altered practice, which varied from time to time, between hospitals, and even from midwife to midwife. For several decades, the importance of being together as a couple during the entire process of becoming parents has been recognized. Not being able to do so disrupts the creation of the family unit and may cause both short- and long-term negative consequences. An important future research area might be to investigate the longer-term impact on families of the restrictions at this time in their lives. Furthermore, questions such as how infection prevention and the relational aspects of caring might be integrated, and if so, how the inherent tensions could be reconciled are of importance.

### Strengths and limitations

The strength of this study is that, to our knowledge, few studies have investigated the partners’ experiences with pregnancy, labour, and the postpartum period in general; moreover, as far as we know, this is one of the few studies to investigate partners’ experiences due to the COVID-19 pandemic. The relatively high number of participants might be seen as a strength. However, some of the interviews did not last for more than 30 minutes, so eighteen interviews seemed sufficient for the analysis. We used, Zoom to implement the interviews. Using digital platform might reduce the possibilities of noticing nonverbal signals. Additionally, they were recruited from all parts of the country, and the participants differed in parity, which could reveal different multifaceted experiences. The participants found the topic quite interesting and were willing to share their experiences. A limitation of this study was that we used the snowball method in collecting data. The researchers started the recruitment process by asking people of their own acquaintance, which may result in a sample is relatively uniform. Most of the participants have higher education degrees and freely expressed their views—they may thus have been more empowered in relation to and more critical towards the organization of the healthcare system during the COVID-19 pandemic. Another potential limitation is related to the authors’ perceptions, which could affect the analysis of the data, as with any study of this kind. Prior to this study, the authors had some familiarity with co-parents’ experiences during the COVID-19 pandemic. Based on these experiences, we developed the idea of pursuing these assumptions through systematic research. This knowledge might have informed both the questions asked and the interpretations. However, we have tried to maintain reflexivity about personal experiences that could have influenced the analysis through maintaining an ongoing, critical discussion within the research team.

## Conclusion

The co-parents were deprived of doing what they considered to be their most important job—namely, to support, comfort, and be present for their partner during pregnancy and childbirth. However, it was also important for the participants to partake in the whole process in their own transition to parenthood, to be able to create their own identity as co-parent.

By not being allowed into the health facilities, the co-parents experienced not being considered to be an equal part of the team in creating a family, leading to feelings of worry, anger, frustration, and even shame. This exclusion was partly a result of restrictions due to infection prevention during the COVID-19 pandemic, but it is worth noting that that even before the COVID-19 pandemic, healthcare providers seem to have been unsuccessful in involving co-parents in pregnancy and postnatal care. The health authorities’ decision to exclude co-parents from being physically present during the process of becoming parents requires analysis and discussion. Questions must be asked regarding the ways restrictions due to COVID-19 reflect the healthcare authorities and healthcare workers’ views of the importance of the non-birthing parent’s participating role in pregnancy, birth, and the postnatal period.

## Data Availability

The dataset for this study is not open access but can be made available (in Norwegian) upon request to the Principal Investigator (FO) and according to ethical approval.

## Note

1. “Active labour” refers to the point when the labour has progressed to a certain opening of the cervix and there are well-established contractions. At this point, the process of labour is expected to speed up and lead to the birth of the baby.
